# Back to the future—revisiting Skylab data on ocular counter-rolling and motion sickness

**DOI:** 10.3389/fphys.2023.1303938

**Published:** 2023-11-21

**Authors:** Gilles Clément, Timothy R. Macaulay, Sarah C. Moudy, Olga Kuldavletova, Scott J. Wood

**Affiliations:** ^1^ KBR, Houston, TX, United States; ^2^ Aegis Aerospace, Houston, TX, United States; ^3^ INSERM U1075 COMETE, University of Caen Normandy, Caen, France; ^4^ Neuroscience Laboratory, NASA Johnson Space Center, Houston, TX, United States

**Keywords:** space motion sickness, ocular counter-rolling, Coriolis sickness, parabolic flight, asymmetry

## Abstract

In the early 1970s, nine astronauts participated in missions to the Skylab space station. During two preflight testing sessions at the Naval Aerospace Medical Research Laboratory in Pensacola, the amplitudes of their ocular counter-rolling (OCR) during body tilts were assessed to determine if their vestibular functions were within normal ranges. We recently re-evaluated this data to determine asymmetry of each astronaut’s OCR response and their OCR slope from sigmoid fits during static leftward and rightward body tilts, which we then compared with their Coriolis sickness susceptibility index (CSSI) on the ground, their motion sickness symptom scores during 0 g maneuvers in parabolic flight, and the severity of the symptoms of space motion sickness (SMS) they reported during their spaceflights. We arranged the astronauts in rank order for SMS severity based on the SMS symptoms they reported during spaceflight and the amount of anti-motion sickness medication they used. As previously reported, the OCR amplitudes of these astronauts were within the normal range. We determined that the OCR amplitudes were not correlated with SMS severity ranking, CSSI, or motion sickness symptoms experienced during parabolic flight. Indices of asymmetry in the OCR reflex were generally small and poorly correlated with SMS scores; however, the only subject with a high index of asymmetry also ranked highly for SMS. Although OCR slope, CSSI, and motion sickness symptoms induced during parabolic flight were each only moderately correlated with SMS severity ranking (rho = 0.41–0.44), a combined index that included all three parameters with equal weighting was significantly correlated with SMS severity ranking (rho = 0.71, *p* = 0.015). These results demonstrate the challenge of predicting an individual’s susceptibility to SMS by measuring a single test parameter in a terrestrial environment and from a limited sample size.

## Introduction

May 2023 marked the 50th anniversary of the first crewed Skylab mission (Skylab-2), which lasted 28 days (from 25 May 1973, to 22 June 1973). The second Skylab mission (Skylab-3) lasted 56 days (from 28 July 1973, to 25 September 1973), and the third (Skylab-4) lasted 84 days (from 16 November 1973, to 8 February 1974). Nine astronauts participated in these missions, three per mission. Medical experiments were performed on these Skylab astronauts before, during, and after their missions to assess physiological responses of exposure to weightlessness, whereas crewmembers of the previous Apollo and Gemini mission were assessed only before and after their missions (Johnston, 1977).

Dr. Ashton Graybiel and his colleagues from the Naval Aerospace Medical Research Laboratory (NAMRL) in Pensacola, FL conducted an extensive investigation of each Skylab astronaut’s vestibular system. In the so-called *Experiment M-131–Human Vestibular Function*, a rotating chair was used to study their vestibular function and their susceptibility to motion sickness on board Skylab (Miller and Graybiel, 1973; Graybiel et al., 1977; Lackner and DiZio, 2006). The preflight tests included a measure of ocular counter-rolling (OCR) during static body tilt to the right and the left to assess the sensitivity of the otolith organs’ response to linear acceleration (Diamond and Markham, 1989). The Coriolis sickness susceptibility index (CSSI) test was also performed before flight. CSSI is calculated from the number of head movements in four cardinal directions the astronauts were able to complete while they were rotating in a chair at increasing velocity until they developed motion sickness (Miller and Graybiel, 1974). Each crewmember also reported motion sickness symptoms they experienced during 0 g parabolic maneuvers and reported the symptoms and the anti-motion sickness medications they took during different phases of the Skylab missions (Graybiel et al., 1975; Graybiel et al., 1977). Our retrospective analysis used the original OCR data set, which was recently identified in the archives of Drs. Jerry Homick and Millard Reschke at the NASA Johnson Space Center.

Structural differences in the right and left otolith organs can lead to slightly different sensitivities to vestibular sensing. Normal, healthy individuals in a 1 g gravitational environment use central processes to compensate for this naturally occurring peripheral vestibular asymmetry. Some authors have suggested that bilateral asymmetry in OCR is associated with susceptibility to motion sickness (Von Baumgarten and Thumler, 1979; Lackner et al., 1987; Markham and Diamond, 1993; Nooij et al., 2011; Sugawara et al., 2021). A recent study using inner ear magnetic resonance imaging determined that individuals who were highly susceptibility to motion sickness had larger morphological asymmetry of the bilateral vestibular organs (Harada et al., 2021).

**FIGURE 1:**
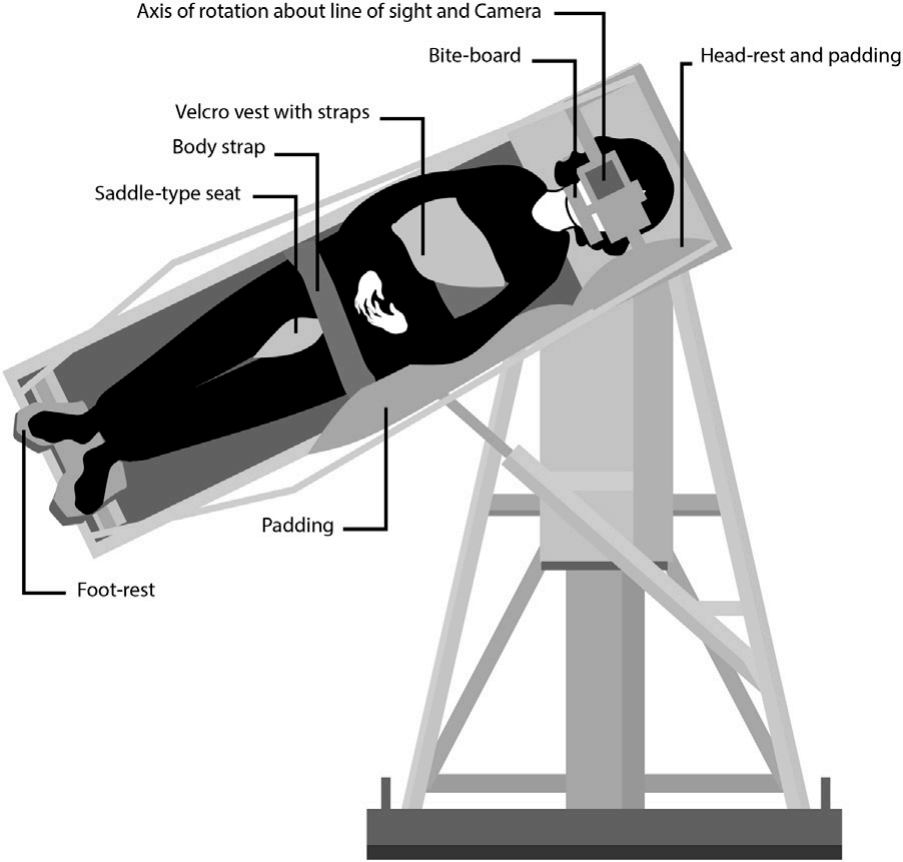
Diagram showing the counter-rolling test device at the Naval Aerospace Medical Research Laboratory in Pensacola, FL. The subjects were tilted up to 64 deg to the side around an axis that was aligned with their right or left eye. A camera placed on a platform in front of the subject’s face took photographs of the eye for offline measurements of ocular counter-rolling. Credit: Olga Kuldavletova [adapted from Miller and Graybiel (1972)].

Lackner et al. (1987) examined asymmetric otolith function in healthy subjects using the same device that was used in the Skylab studies (Figure 1) to determine if OCR asymmetry is associated with increased susceptibility to motion sickness during exposure to various levels of gravito-inertial acceleration. The average indices of OCR asymmetry in the highly susceptible group (42 of 71 subjects) were approximately twice that of the low and the moderate susceptible groups. Although the indices of OCR asymmetry did not predict susceptibility in all cases, this study suggested that otolith asymmetries for some individuals, which manifest as OCR during static roll tilt testing in 1g, may be associated with susceptibility to motion sickness in altered gravito-inertial environments. Therefore, we conducted this retrospective analysis to determine whether correlations existed between the Skylab astronauts’ preflight OCR asymmetry during leftward and rightwards body tilts and the severity of their space motion sickness (SMS) symptoms during flight. A secondary objective was to examine how the new OCR parameters derived from a sigmoidal fit to the OCR data relate to other preflight susceptibility tests described above (CSSI and motion sickness symptoms during parabolic flight), and how these parameters relate to the SMS severity.

## Methods

### Participants

The nine Skylab astronauts were all male, aged 41.3 ± 2.0 years (mean ± SD), who were selected through extensive screening procedures (Santy, 1994). Seven of the astronauts flew for the first time on their Skylab mission, two astronauts had participated in a previous Apollo mission that included a Moon landing, and one of these two astronauts had also participated in a Gemini mission. Individual characteristics of the nine Skylab astronauts were reported in Johnson and Dietlein (1977).

All the Skylab astronauts elected to participate in *Experiment M-131—Human Vestibular Function*. This experiment was performed, like all the other medical experiments on board Skylab, in accordance with the ethical standards established by the 1964 Declaration of Helsinki. All subjects provided written, informed consent before participating in the study (Johnston, 1977).

### Experimental protocol

The aim of the Skylab M-131 experiment was to measure vestibular responses in astronauts while they were weightless during orbital flight and to compare these responses to measurements taken before and after flight. The parts of the M-131 experiment that related to susceptibility to motion sickness included a) evaluating the astronaut’s susceptibility to a variety of motion sickness stressors, including maneuvers during 0 g in parabolic flight and Coriolis, cross-coupled angular accelerations during pitch and roll head movements while being rotated about an Earth-vertical axis; b) measuring the amplitude of OCR during static body tilt in roll relative to gravity; and c) grading the severity of SMS using diagnostic criteria (Miller and Graybiel, 1973; Graybiel et al., 1977).

Preflight functional tests of the astronauts' vestibular organs using caloric nystagmus and head rotation stimuli revealed no abnormalities. A postural equilibrium test that required the astronauts to maintain postural equilibrium on narrow metal rails with their eyes open or closed indicated that their responses were within the normal range (Homick and Reschke, 1977). Other than the activities indicated above, none of the Skylab astronauts underwent a specific vestibular training or vestibular desensitization program.

During tests to grade the astronauts’ susceptibility to motion sickness on Earth, the astronauts sat in a chair that rotated at angular velocities up to 30 rpm and were asked to execute 90-deg head movements (front, back, left, and right). The CSSI was then determined for each subject by multiplying an E-factor related to the rotation velocity and the number of head movements required to provoke a severe malaise (Miller and Graybiel, 1970). CSSI scores above 15 are generally considered to be in the low susceptibility range (Miller and Graybiel, 1974). The severity of motion sickness symptoms during 0 g parabolic maneuvers were also reported (Graybiel et al., 1977). Symptoms category included nausea, epigastric discomfort, skin color, cold sweating, increased salivation, drowsiness, and headache (Graybiel et al., 1968).

OCR was evaluated at the NAMRL from August 1972 to April 1973. Subjects assumed a semi-standing position in the counter-rolling test device, with their weight distributed between a saddle-type seat arrangement and an adjustable foot-rest platform (Figure 1). Their heads were maintained in place using a locked headrest and bite-board assembly. A camera recording system was placed on a platform in front of their face. The subjects were shifted sideways until the center of the pupil of their right or left eye was aligned with the optic axis of the camera and the rotation axis of the test device. Subjects were tilted 17.5, 25, 39, 50, and 64 deg from upright, alternately to the right and to the left (Miller, 1962). No OCR measurements were performed post-flight.

**FIGURE 2:**
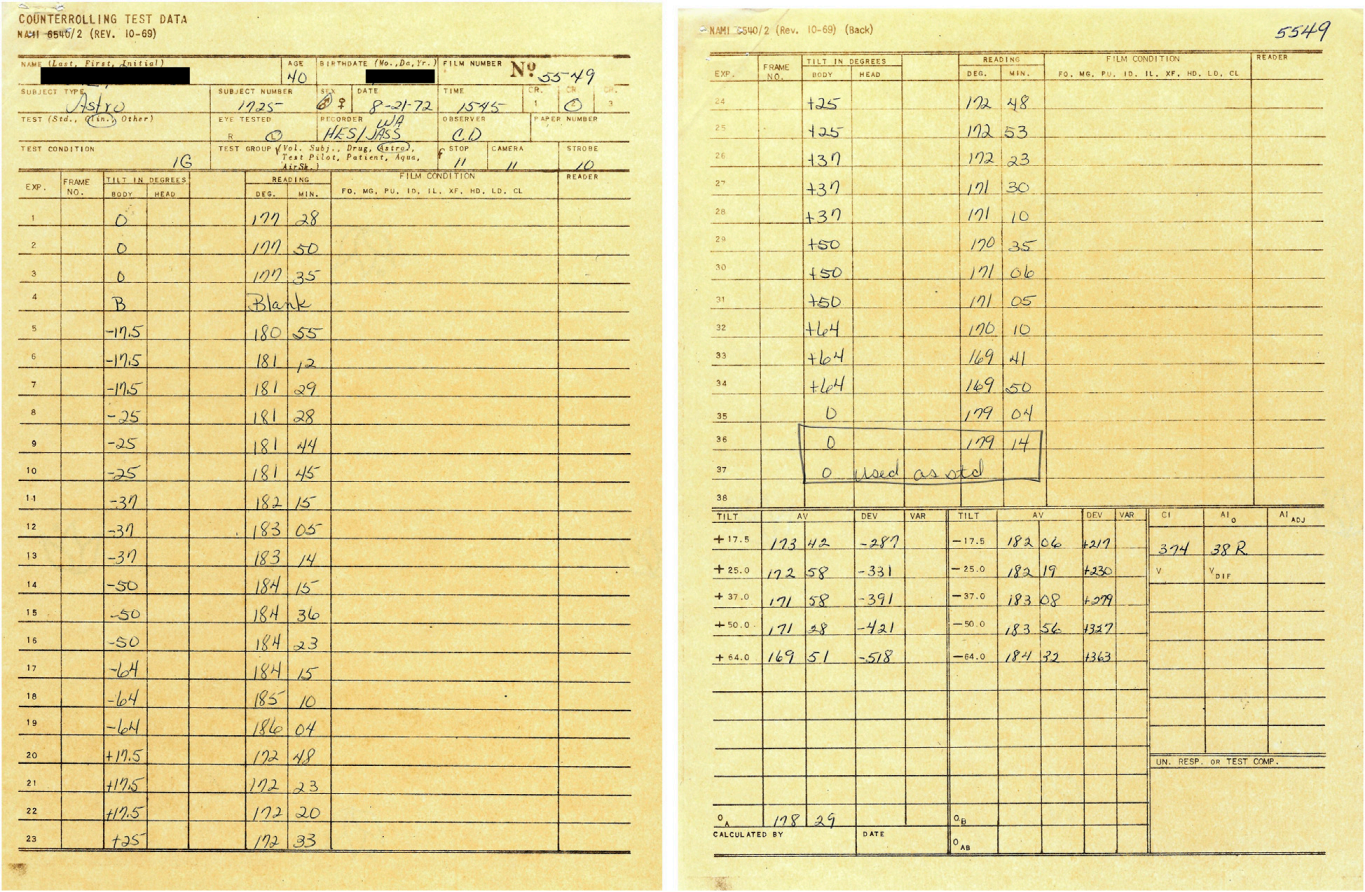
Example of worksheet used during collection of the ocular counter-rolling test data at the Naval Aerospace Medical Research Laboratory. The name and date of birth of the astronaut-subject have been masked.

Four trials were performed during two sessions. During the first session, either the right or the left eye was recorded during body tilt to the right and to the left. The other eye was tested the following day. An example of the datasheet used during one session is shown in Figure 2. Three photographs of the eye were taken at each body tilt angle for subsequent analysis of eye position based on natural iris landmarks. The recorded positions of the eye roll during the initial and the terminal upright body positions were used as the baseline (zero) position to which all other OCR measurements were related (Miller, 1962). Because the OCR of the right and the left eye were assessed on separate days, it was not possible to measure ocular torsional disconjugacy, which appears to be associated with a history of SMS (Markham and Diamond, 1993).

**FIGURE 3:**
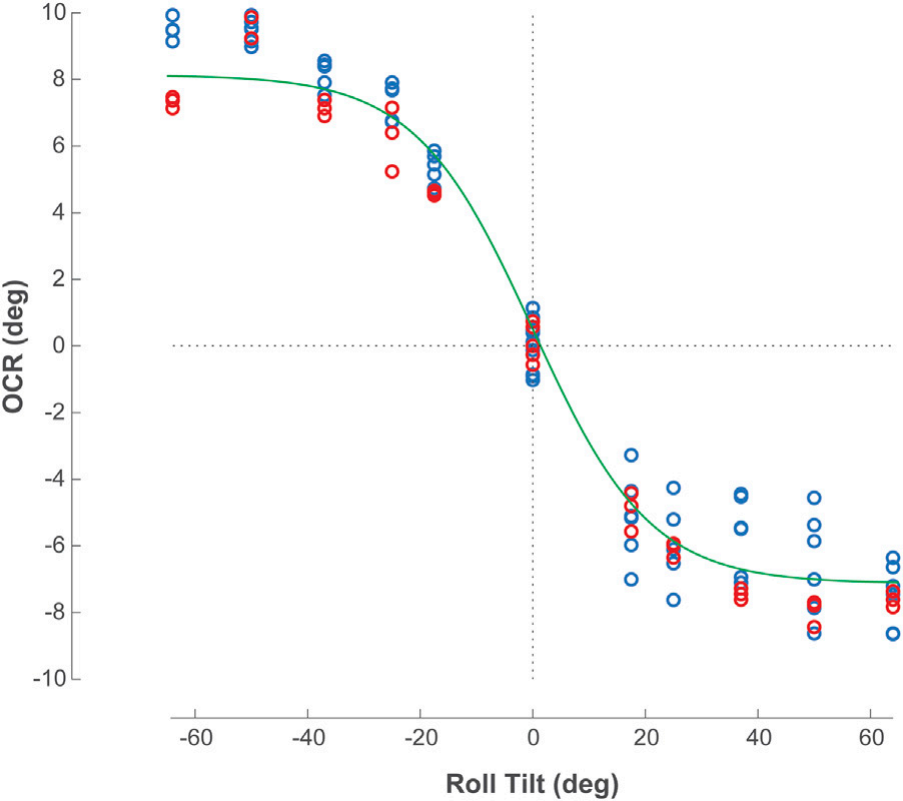
Amplitude of ocular counter-rolling (OCR) of a typical Skylab astronaut during actual body tilts to the right and to the left. Body tilt to the right (positive values) generated a counterrotation of the eye to the left (negative values). Multiple measurements of OCR were taken for each eye at each body tilt position. Red symbols: measures of OCR of the right eye; blue symbols: measures of OCR of the left eye. Source: Naval Aerospace Medical Research Laboratory, Pensacola, FL. The sigmoidal curve fit (green line) using all OCR values was based on a three-parameter model to obtain leftward and rightward OCR and the slope around the upright orientation (Roll Tilt = 0°).

OCR index was previously calculated by Graybiel et al. (1977) as half the maximum eye roll amplitude when tilted right and left. However, this measure does not account for the variations in OCR amplitude across various roll tilt angles nor the sensitivity of the reflex to tilts near the upright orientation. Using the original OCR data collected at the NAMRL, we calculated OCR parameters in each astronaut. Data obtained from trials of each eye at all angles of roll tilt were combined and fit with a sigmoid function in MATLAB (version R2022b, The MathWorks, Inc.) using three free parameters: maximum OCR during rightward tilt, maximum OCR during leftward tilt, and slope at the zero crossing (i.e., OCR slope, see sample in Figure 3). Similar to the method used by Lackner et al. (1987), the otolith asymmetry ratio was then computed by taking the ratio of the larger to the smaller ocular counter-rolling responses for left tilts and right tilts, subtracting 1, and then multiplying by 100.

The occurrence and frequency of SMS the Skylab astronauts experienced and their use of medication to counter the symptoms of SMS have been previously reported (Graybiel et al., 1975; 1977). Studies have commonly reported high degrees of inter-subject variability in SMS (Davis et al., 1988; Reschke et al., 2017). To examine how the preflight motion sickness susceptibility and OCR indices related to SMS data in this limited sample set, we rank ordered the astronauts according to SMS susceptibility based on the sum of symptom points reported by Graybiel et al. (1977) across mission phases (before docking, after docking, and during flight days 1–3). If astronauts had the same number of symptom points, the number of SMS medications they took were used to assign the ranking. Relationships between the various parameters were analyzed using non-parameter Spearman rank correlation (SPSS Statistics, v29, IBM Corp.).

## Results

The nine Skylab astronauts’ preflight CSSI, OCR indices, and symptom scores during 0 g parabolic flight have been previously published by Graybiel et al. (1977) and are shown in Table 1. Six of the astronauts had CSSI scores that indicated low susceptibility: five had scores in the top 80% of normal responses, and one (Subject H) had scores in the top 90% (Miller and Graybiel, 1974). Symptoms reported during 0 g maneuvers in parabolic flight ranged between 2 and 16, again most of this cohort were in the low susceptibility range.

**TABLE 1:** Values for Coriolis sickness susceptibility index (CSSI), motion sickness symptoms during 0 g maneuvers in parabolic flight, ocular counter-rolling (OCR) index, OCR asymmetry, OCR slope and space motion sickness (SMS) for the 9 Skylab astronauts (A-I).

		Skylab-2			Skylab-3			Skylab-4		
		A	B	C	D	E	F	G	H	I
CSSI^a^		10.2	19.8	8.2	23.1	19.2	26.4	7.5	52.8	8.9
Parabolic 0 g symptoms^a^		4	2	4	15	4	4	16	8	8
OCR Index (deg)^a^		2.63	6.23	5.00	6.08	5.53	2.63	8.23	4.23	4.35
OCR Asymmetry		13.9	25.4	15.0	21.1	54.0	17.5	9.5	4.5	19.5
OCR Slope (from sigmoid fit)		0.034	0.025	0.033	0.028	0.027	0.035	0.034	0.048	0.016
SMS Symptoms^a^	Predock	0	0	0	0	1	0	1	3	0
	Postdock	0	0	0	0	1	0	1	2	0
	FD1	0	0	0	2	3	3	0	1	0
	FD2	0	0	0	2	1	2	1	1	0
	FD3	0	0	0	0	1	1	1	1	0
SMS Medication^a^	Predock	0	0	1				2	2	2
	Postdock	0	0					3	3	3
	FD1	0	0							
	FD2	0	0		2	2	2	3	2	3
	FD3	0	0			3		1	2	1
SMS Rank		1	1	3	5	8	7	6	9	4

Predock: inside the Apollo module before docking with Skylab; Postdock: inside the Apollo module after docking with Skylab. FD = flight day in Skylab; SMS Symptoms: 1 = slight, 2 = moderate, 3 = severe. The values in the SMS medication rows represent the number of anti-motion sickness medication taken by each astronaut.

^a^ Shaded cells indicate original data published in Graybiel et al. (1977).

As previously reported (Graybiel et al., 1975; Graybiel et al., 1977), none of the Skylab-2 crewmembers experienced SMS. Astronaut E of the Skylab-3 crew experienced motion sickness within an hour of transition into orbit (before and after docking). All three Skylab-3 astronauts experienced motion sickness during the first 3 days of spaceflight. These astronauts obtained relief by avoiding head and body movements and by a using a combination of Scopolamine (0.35 mg) and Dexedrine (5.0 mg). All three astronauts of the Slylab-4 crew took prophylactic medication before entering the Skylab station and continued to do so on flight days 2 and 3. This medication included a combination of Scopolamine (0.35 mg) and Dexedrine (5.0 mg) or a combination of Promethazine (25 mg) and Ephedrine (50 mg). Astronaut I did not get sick while on board, whereas the other two Skylab-4 astronauts got moderately sick during the first 3 days of spaceflight (Table 1). On and after the sixth day of spaceflight none of all nine astronauts experienced SMS, including when moving their head while on the Skylab rotating chair that generated Coriolis, cross-coupled angular accelerations (Graybiel et al., 1977).

The amplitudes of the OCR in the nine Skylab astronauts were clearly related to the angle of head tilt (i.e., the magnitude of the acceleration vector in the plane of the utricles) and the amplitude of the eye torsional movement. The amplitude of this otolith-mediated eye movement was approximately 10% of the maximum head tilt (Figure 3). Previous tests conducted in 550 normal subjects reported a mean OCR index of 5.73 deg (Graybiel, 1970). Six of the Skylab astronauts (A, C, E, F, H, I) had OCR index values that were lower than normal. However, five of the Skylab astronauts (C, D, E, G, H) had higher OCR amplitudes when tilted to the right, whereas four of the Skylab astronauts (A, B, F, I) had higher OCR amplitudes when tilted to the left (Figure 3; Table 1).

**FIGURE 4:**
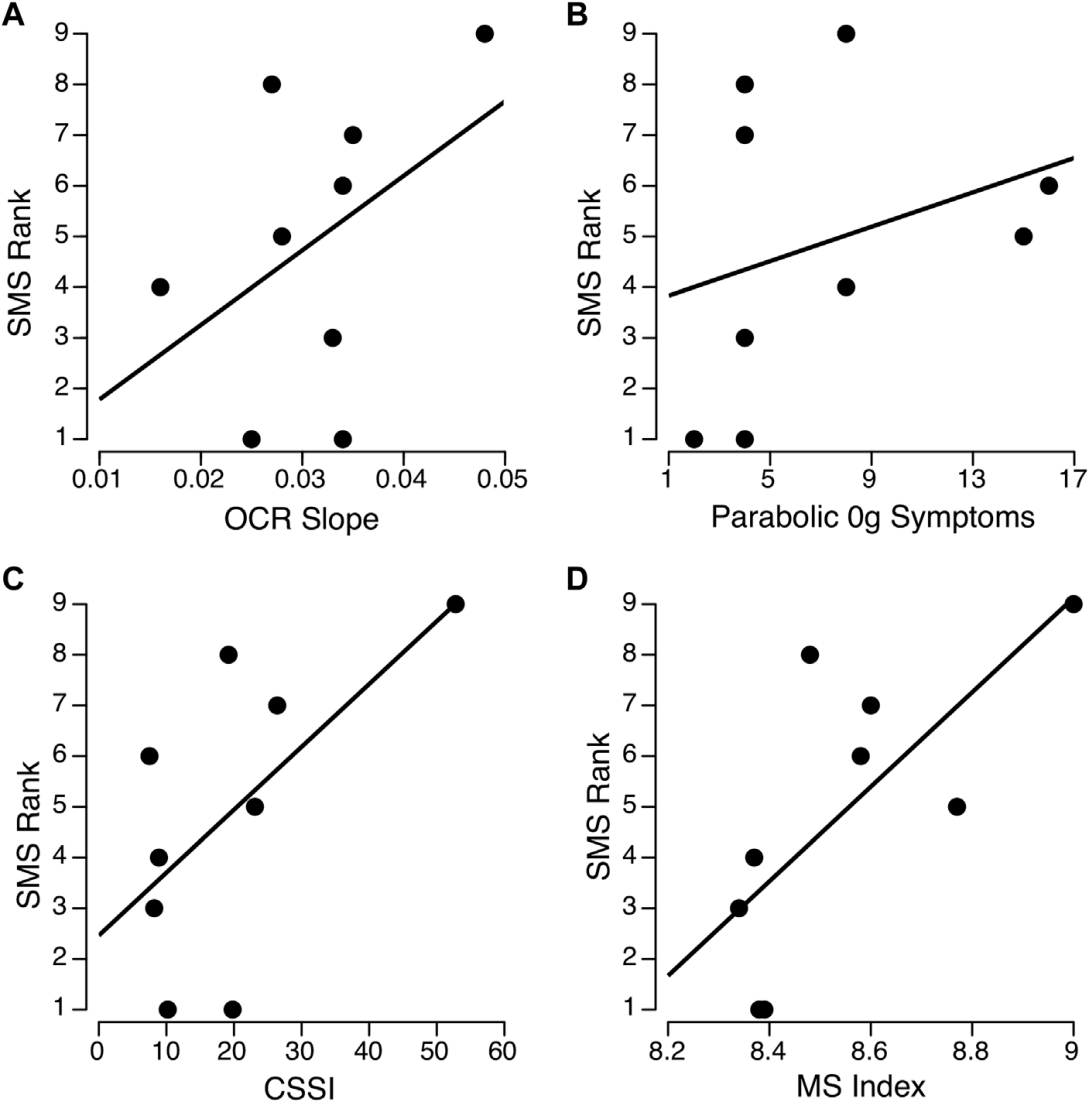
Relationships between ocular counter-rolling (OCR) slope **(A)** motion sickness symptoms during parabolic flight **(B)**, Coriolis sickness susceptibility index (CSSI) **(C)**, and composite motion sickness (MS) index **(D)** with Space Motion Sickness (SMS) ranking for the nine Skylab astronauts. The parabolic and CSSI scores were obtained from Graybiel et al. (1977). Spearman’s rho was between 0.41 and 0.44 across the three separate measures **(A–C)** and 0.71 (*p* = 0.015) for the composite MS index **(D)**.

The new OCR parameters from the sigmoidal fits include the amplitude in each direction and its slope around the upright orientation. The average amplitude from the sigmoidal fits were significantly correlated with the OCR index previously reported by Graybiel et al. (1977) (rho = 0.945, *p* < 0.001). Therefore, we only included the original OCR index in this analysis. As described above, the OCR amplitudes in the left and the right eye from the sigmoidal fits were used to calculate OCR asymmetry using the same convention used by Lackner et al. (1987). Interestingly, OCR asymmetry was negatively correlated with OCR slope from the sigmoidal fits (rho = −0.77, *p* = 0.008). The OCR asymmetry measures were small and not significantly correlated with any of the measures of motion sickness susceptibility. Eight of the 9 astronauts had OCR asymmetries ≤25, consistent with the low susceptibility groups reported by Lackner et al. (1987). The only Skylab astronaut with an OCR asymmetry consistent with the high susceptibility group also had a high SMS rank (8 of 9). The OCR slope from the sigmoidal fit was moderately correlated with SMS ranking (Figure 4A, rho = 0.41, *p* = 0.14).

The correlations between SMS ranking and motion sickness symptoms during 0 g in parabolic flight or CSSI were also moderate but non-significant (Figures 4B, C; Table 2). Given the OCR slope, motion sickness symptoms during 0 g parabolic flight, and CSSI scores were moderately but not significantly correlated with SMS rank, each of these variables were then transformed using a two-step approach described by Templeton (2011). This process involved 1) fractional ranking of each variable, and 2) transformation using an inverse distribution function with a normalized mean and standard deviation so that the three parameters (OCR slope, CSSI, and motion sickness symptoms during parabolic flight) could be averaged to compute a composite motion sickness index. The resulting motion sickness index, which comprises the contributions of all three variables, significantly correlated with SMS ranking (Figure 4D; Table 2, rho = 0.72, *p* = 0.015).

**TABLE 2:** Results of the non-parametric correlation analysis (* indicates *p* < 0.05).

		OCR index	OCR asym	OCR slope	CSSI	Parabolic	MS index
OCR index	rho						
	sig (1-tail)						
OCR asym	rho	0.32					
	sig (1-tail)	0.20					
OCR slope	rho	−0.51	−0.77 *				
	sig (1-tail)	0.08	0.008				
CSSI	rho	−0.37	0.05	0.37			
	sig (1-tail)	0.17	0.45	0.17			
Parabolic	rho	0.26	−0.42	0.15	−0.16		
	sig (1-tail)	0.25	0.13	0.35	0.34		
SMS rank	rho	−0.09	−0.16	0.41	0.44	0.41	0.71 *
	sig (1-tail)	0.41	0.34	0.14	0.12	0.14	0.015

The light shaded areas are plotted in Figure 4.

OCR, ocular counter-rolling; asym: asymmetry; CSSI, Coriolis sickness susceptibility index; SMS, space motion sickness; Parabolic, motion sickness symptoms during 0 g maneuvers in parabolic flight.

## Discussion

This reanalysis of the Skylab data allowed us to investigate relationships between OCR parameters measured during static roll tilt and other preflight susceptibility tests and the Skylab astronauts’ SMS ranking. Given the limitations of the small sample size, caution should be exercised when interpreting these results. However, the inability to identify a significant association between SMS susceptibility and a single measure obtained in a terrestrial environment is consistent with the results of previous studies of larger cohorts (Reschke, 1990). As Lackner et al. (1987) pointed out, otolith asymmetry reflected by the static roll tilt may contribute to SMS but is likely not the only cause of SMS. A significant relationship to SMS ranking was only found after combining the OCR slope, CSSI scores, and motion sickness symptoms induced by parabolic flight, which presumably represent a multitude of contributing factors.

It is important to point out that an association between SMS and otolith asymmetry cannot be ruled out completely because underlying otolith asymmetry that is centrally compensated for in terrestrial conditions (Von Baumgarten and Thumler, 1979) might not be detected during static roll tilt in 1 g. Markham and Diamond (1993) reported that ocular torsional disconjugacy was related to motion sickness susceptibility, but only during exposure to altered gravito-inertial states during parabolic flight. Subjects who experienced motion sickness after altered g-exposure from sustained centrifugation on Earth, i.e., after a transition from 3 g to 1g, were more likely to experience SMS (Nooij et al., 2007). However, the authors noted that subjects who are susceptible to motion sickness during centrifugation had only a marginally higher degree of OCR asymmetry than subjects who were not susceptible (Nooij et al., 2011). Consistent with our composite motion sickness index, a combination of utricular and semicircular canal parameters better predicted the subjects who are susceptible motion sickness during centrifugation.

OCR is considered to reflect mainly utricular responses to interaural acceleration accompanied with lateral head tilt (Clarke et al., 2003; Otero-Milan et al., 2017). One limitation of the OCR test during body tilt is that it is a bilateral otolith stimulation, i.e., the gravitational acceleration stimulus is equivalent for both the right and left otolith organs. At present the best practical approaches for testing unilateral otolith function are measuring OCR during unilateral centrifugation (Clarke et al., 2003; Wuyts et al., 2003), assessing ocular vestibular evoked myogenic potentials (VEMP) to test utricular function, and measuring cervical VEMP as an indicator of saccule function (Manzari et al., 2012).

Evidence indicates that OCR during static body tilt or lateral centrifugation decreases after spaceflight, particularly after long-duration space missions (Hallgren et al., 2016; Reschke et al., 2018; Schoenmaeker et al., 2022). Six of the Skylab astronauts’ preflight OCR amplitudes were lower than of the normal population. The low OCR amplitudes and OCR asymmetry values limited our ability to find and an association between the M-131 data and the SMS ranking.

If central asymmetry in otolith function is unmasked by exposure to weightlessness, then asymmetry may be detected after flight. Clarke and Schonfeld (2015) showed that OCR asymmetry, subjective visual vertical during unilateral centrifugation, and cervical VEMP (which reflects saccular function) increased after spaceflight relative to preflight baseline values and returned to baseline levels within 10 days. On landing day, the response from one vestibular labyrinth was equivalent to preflight values, whereas the other labyrinth had considerable discrepancy. Unfortunately, the OCR test during body tilt used in the Skylab M-131 experiment cannot discriminate such asymmetry between the vestibular organs.

Although Lackner et al. (1987) cautioned that OCR asymmetry was insufficient to predict an individual’s susceptibility to motion sickness during parabolic flight, they demonstrated group mean differences in OCR asymmetries could predict motion sickness. Previous attempts to predict an individual’s SMS from their preflight susceptibility to motion sickness have also been elusive, although group differences suggest some relationships. For example, Homick et al. (1987) found that 67% of SMS susceptible crewmembers had CSSI scores below the mean (i.e., were more susceptible to CSSI) whereas only 40% of non-susceptible crewmembers were below the mean. The authors concluded that a single ground-based test parameter or procedure was inadequate to predict SMS susceptibility and recommended the use of a composite or weighted score. The association between SMS ranking and our Spearman correlation rho values for OCR slope, CSSI, or motion sickness symptoms during parabolic flight were greater than 0.4 but were non-significant. However, the composite motion sickness index that averaged the three parameters did result in a significant association to SMS rank in this limited sample set.

OCR is an important measure of otolith utricular function. It is possible, however, that asymmetry in saccular responses is more closely associated with motion sickness susceptibility than is asymmetry in utricular responses. After assessing both ocular VEMP (which reflects utricular function) and cervical VEMP (which reflects saccular function), Singh et al. (2014) reported that individual susceptibility to motion sickness is associated not only with asymmetry of utricular functional but also with asymmetry of saccular functional.

Because OCR gain is very low, it is inadequate to compensate for head tilt. By contrast the modulation of neck, trunk, and muscle musculature by the otolith-spinal pathways is very important for postural control. Lackner and Dizio (2006) suggested that individuals could centrally compensate for asymmetric OCR using these otolith-spinal pathways. Central compensatory effects could also occur in individuals with unbalanced peripheral inputs from the otolith organs, which could be due to differences in otoconial mass between the paired otolith organs.

Given that a combination of many motion types can cause motion sickness in real-life situations, predicting susceptibility to motion sickness from laboratory experiments has some limitations. SMS remains a persistent problem during spaceflight missions, both when astronauts enter the weightless environment and when they return to Earth after long-duration missions. The Skylab M-131 experiment clearly showed that astronauts were no longer susceptible to motion sickness when exposed to Coriolis, cross-coupling stimulation on or after the sixth day of their spaceflight. On return to Earth, they were less susceptible to this type of stimulation than they were before flight and remained so for several weeks (Lackner and Dizio, 2006). Therefore, further research is needed to better understand motion sickness susceptibility and vestibular adaptation.

## Data Availability

The original contributions presented in the study are included in the article/supplementary materials, further inquiries can be directed to the corresponding author.
